# Sustained Circadian Rhythms in Continuous Light in *Synechocystis* sp. PCC6803 Growing in a Well-Controlled Photobioreactor

**DOI:** 10.1371/journal.pone.0127715

**Published:** 2015-06-01

**Authors:** Pascal van Alphen, Klaas J. Hellingwerf

**Affiliations:** 1 Molecular Microbial Physiology Group, Swammerdam Institute for Life Sciences, University of Amsterdam, Amsterdam, The Netherlands; 2 Photanol BV, Amsterdam, The Netherlands; Universidade Federal de Vicosa, BRAZIL

## Abstract

The cyanobacterial circadian clock has been well-studied and shown to be both robust and a dominant factor in the control of gene expression in *Synechococcus elongatus* PCC7942. In *Synechocystis* sp. PCC6803, the circadian clock is assumed to function similarly, yet appears to control transcription to a far lesser extent and its circadian rhythm was reported to not be sustained, or at least rapidly damped, under continuous illumination. One of the feedback loops that governs the clock in *S*. *elongatus* in addition to the core oscillator, i.e., the transcriptional-translation regulation loop hinging on KaiC-dependent expression of *kaiBC*, appears to be missing in *Synechocystis*, which would account for this difference. Here, we show that the clock in *Synechocystis* fulfills all criteria of a circadian clock: 1) a free-running period of approximately 24 h, 2) temperature compensation, and 3) being able to be entrained. A remarkably stable rhythm is generated despite the fact that the organism grows with a doubling time of less than 24 h in a photobioreactor run in turbidostat mode. No damping of the free-running circadian oscillation was observed in 2 weeks, suggesting that the clock in individual cells stays synchronized within a culture despite the apparent lack of a transcriptional-translation regulation loop. Furthermore, the dependence of chlorophyll synthesis on the presence of O_2_ was demonstrated.

## Introduction

Many organisms have a circadian timing mechanism to cope with daily fluctuations in light and nutrient availability and temporally separate mutually incompatible processes [1]. The cyanobacteria are the only known phylum of prokaryotes to have a circadian clock, though there is evidence for the presence of circadian rhythms in other Bacteria [2] and Archaea [3]. Circadian clocks must fulfill three criteria to be called as such: 1) a period of approximately 24 h in the absence of environmental cues (the so-called free-running period); 2) compensation for environmental changes, most prominently temperature, to keep this period at approximately 24 h and 3) the ability to be entrained by environmental cues (zeitgebers) and maintaining its phase relationship with the zeitgeber [4,5].

In *Synechococcus elongatus* PCC7942 (*Synechococcus*), a free-running, temperature-compensated period of approximately 24 h was observed for many physiological phenomena, including gene expression and chromosome compaction [6–9]. This showed that a prokaryote can contain a fully functional circadian clock and *Synechococcus* has since become the model organism to study the cyanobacterial circadian clock [10]. The core oscillator of this circadian clock is a posttranslational regulation mechanism that has been shown to consist of just three proteins, KaiA, KaiB and KaiC, and is even able to generate a rhythmic phosphorylation/dephosphorylation cycle of KaiC when reconstituted *in vitro* [11]. In addition to the core oscillator, a transcriptional-translational regulation (TTR) loop couples the phosphorylation state of KaiC to the transcription of *kaiBC*, thereby introducing a negative feedback loop in which KaiC controls its own production. This TTR loop has been shown to be particularly important for maintaining synchrony within a population [12,13]. Even though homologs of cyanobacterial clock genes have been found in multiple Bacteria and Archaea, complete *kaiABC* gene clusters are only found in cyanobacteria [14,15].

A cyanobacterium that is of particular interest is *Synechocystis* sp. PCC6803 (*Synechocystis*). This is the commonly used model organism in photosynthesis research due to its ability to heterotrophically grow on glucose, even in almost constant darkness [16]. What makes it interesting for clock research is that in addition to a *kaiABC* gene cluster (*kaiAB1C1*), it contains an extra *kaiBC* operon (*kaiB2C2*) and another copy of both *kaiB* (*kaiB3*) and *kaiC* (*kaiC3*), but appears to be missing the promoter in-between *kaiA* and *kaiB1C1* as in *Synechococcus* [17]. The bioluminescence technique of expressing luciferase under the control of a clock-regulated promoter, first applied to *Synechococcus*, yielded similar results in *Synechocystis*, indicating a functional circadian clock [18,19]. However, the resulting rhythm was much more rapidly damped than in *Synechococcus* [18]. Of note is the observation that dark-adapted *Synechocystis* growing in heterotrophic conditions showed a circadian rhythm in continuous darkness (DD), but was unable to phase-reset, due to rapid damping of bioluminescence after a light pulse [19]. As the functioning of the bioluminescence reporter system also depends on other factors besides promoter activity, it is possible that these were effects of the conditions chosen rather than of the circadian clock of *Synechocystis*. Additionally, it should be noted that the bioluminescence experiments were done with bacterial colonies on solid medium, which makes it challenging to precisely control the physiology of all cells involved.

Circadian rhythms of transcript levels of *Synechocystis*, cultivated in liquid medium, were investigated by Kucho et al. in 2005 and recently by Beck et al. in 2014. Kucho et al. showed circadian oscillation of 2–9% of the genes in continuous light (LL) after entrainment by a single pulse of 12 h darkness [20]. Re-analysis of the data in a study comparing *Synechococcus* with *Synechocystis* showed an even lower percentage of significantly oscillating transcripts [21]. The recent study of Beck et al., however, showed no sustained oscillation in either continuous light or darkness, and only weakly oscillating transcripts in a 12 h/12 h light/dark regime (LD) [22]. Whether the oscillation damped over time in either study was not investigated, given that sampling was only done over 48 h. These findings seem quite divergent, which can possibly be explained by the experimental conditions selected. In the work of Kucho et al., a continuous culturing system was used, whereas Beck et al. used a batch culture. Other than that, however, culturing conditions (medium, illumination, temperature) were similar. Kucho et al. reported a circadian rhythm in growth rate, in addition to that found in various transcript levels, suggesting that despite the low number of oscillating genes identified, a free-running phenotype was observed. Conversely, Beck et al. observed very slow growth with no apparent rhythm. In contrast to *Synechocystis*, long-term sustained circadian oscillation has been reported in *Synechococcus* [23].

Here, we show that sustained, temperature-compensated free-running rhythms can be induced in *Synechocystis* under precisely controlled growth conditions, as evidenced by oscillations in growth rate, chlorophyll content and indirectly through dissolved oxygen and carbon dioxide levels, pH, and chlorophyll and phycobilisome fluorescence excitation.

## Results and Discussion

### The circadian clock in *Synechocystis* sp. PCC6803

**Fig 1:**
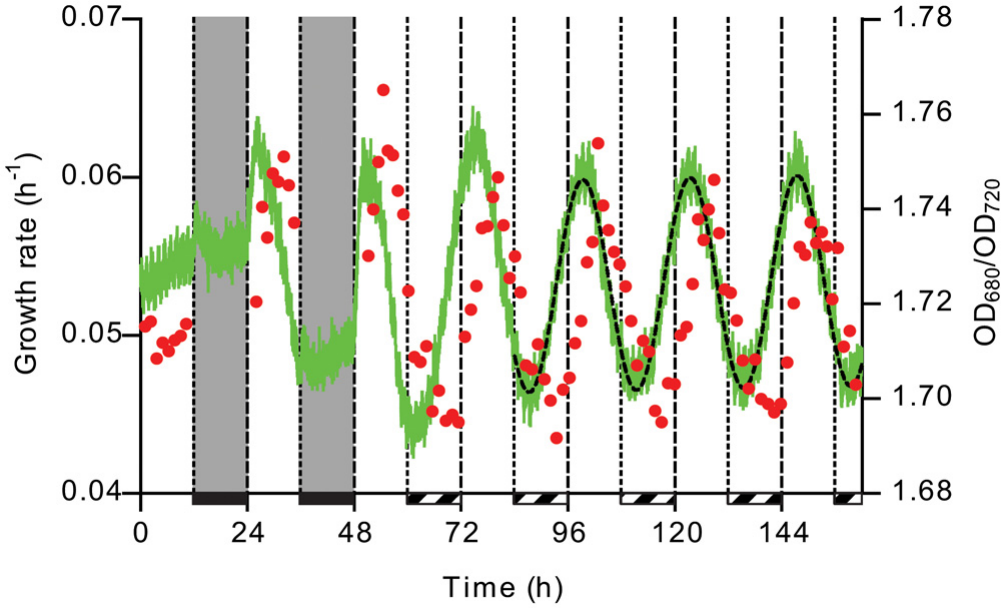
Entrainment and free-running of a *Synechocystis* sp. PCC6803 culture in continuous culture in a photobioreactor. An unsynchronized culture was used to inoculate the photobioreactor approximately 140 h before entrainment, of which the last 12 h in continuous light are shown as the first 12 h. The culture was entrained by two periods of 12h/12h light/dark and subsequently subjected to continuous light. Dark periods are indicated by a grey background and solid dark bars. Subjective ‘night’ in continuous light is indicated by striped bars. Shown are the growth rate (red circles, left axis), the ratio of OD_680_/OD_720_ (green line, right axis) as measured by the photobioreactor, including the fit thereof (dashed line, right axis). Growth rate was calculated from the OD_720_ measured by an integrated photocell in-between pump events using *μ* = (Δln(OD_720_))/Δ*t*. Growth rate data points are plotted in the middle of each pump cycle.

In order to establish whether or not *Synechocystis* has a circadian clock, a *Synechocystis* culture growing in a photobioreactor (PBR) in turbidostat mode was subjected to 12 h/12 h light/dark (LD) entrainment (Fig 1). Entrainment followed a period of continuous illumination (LL) in which a steady state was reached. During the entrainment period, no growth was observed in the dark and a varying growth rate was observed in the light, which peaked in the afternoon of the subjective ‘day’. After 48 h in LD, a free-running period followed in which oscillation of growth rate with a period of approximately 24 h (i.e. 24.1 ±0.1 h) was maintained at the same phase angle relative to the rhythm of the zeitgeber, indicating that an endogenous time-keeping mechanism operates in this organism. This phase relation held, independent of the time point of the actual day at which the experiment was started.

**Fig 2:**
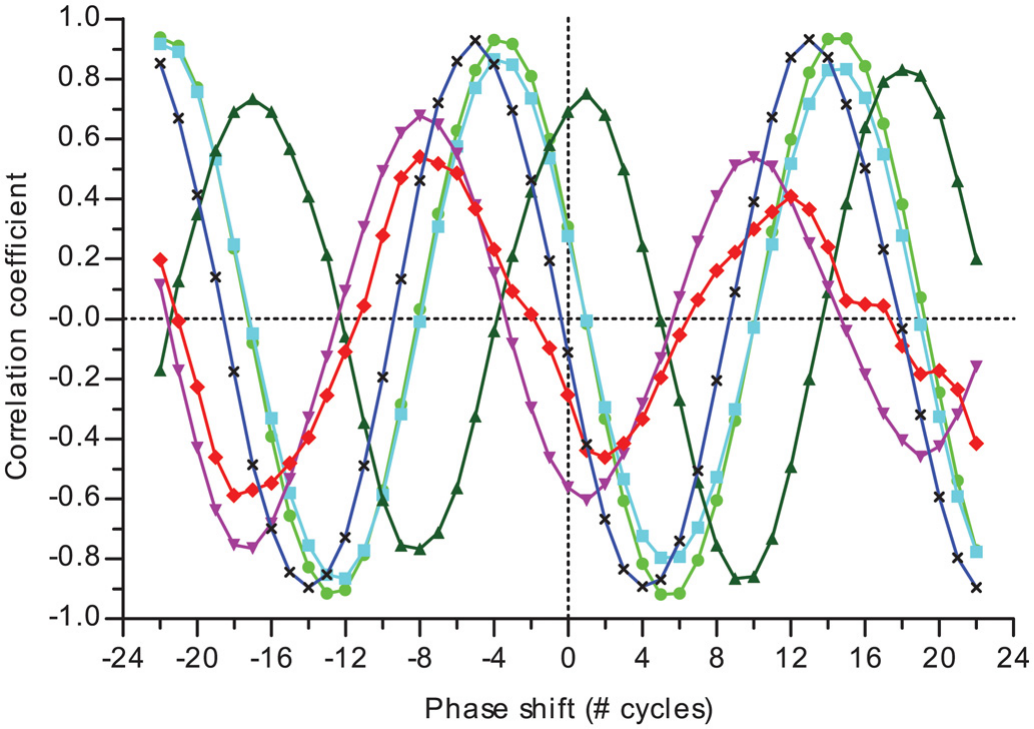
Pearson’s correlation of growth rate with other culture parameters. Most parameters peak together with growth rate in the circadian cycle. Notably, relative chlorophyll content (OD_680_/OD_720_, light green circles), fluorescence emission from chlorophyll excitation (Ft_blue_, blue line with crosses) and phycobilisome excitation (Ft_red_, red diamonds) do not. The data shown here are of the same culture as in Fig 1. A value of 1 indicates a perfect positive linear correlation, 0 the absence of correlation and -1 perfect negative correlation. Note that the phase shift is obtained from sliding along the data points (i.e. growth/dilution cycles), which do not all represent the same time interval due to differences in growth rate. Shown further: pH (cyan squares), dissolved O_2_ (green upward triangles) and dissolved CO_2_ concentration (purple downward triangles).

Other parameters, such as dissolved oxygen (dO_2_), dissolved CO_2_ (dCO_2_) level, pH, PS-II fluorescence emission excited by phycobilisome (Ft_red_) and chlorophyll excitation (Ft_blue_), and OD_680_ displayed similar oscillatory behavior, though not all were at the same phase angle as the growth rate (Fig 2). Particularly, relative chlorophyll content, of which the OD_680_/OD_720_ ratio is a proxy, peaks shortly after subjective dawn; a phase-advance of approximately 6 h with respect to the peak in growth rate in the subjective afternoon. Interestingly, this phase-advance of a quarter of a period with respect to the chlorophyll content approximates the derivative of growth rate. Ft_blue_, originating solely from chlorophyll, is slightly out of phase with the total amount of chlorophyll, indicating that PS-II assembly and (non-) photochemical quenching lags chlorophyll synthesis. Ft_red_, on the other hand, which predominantly originates via the phycobilins, is less-well correlated and peaks even before Ft_blue_, indicating that phycobilisome (PBS) content, state transitions and/or dissipation mechanisms are not or only slightly affected by the circadian clock. However, at 33°C, Ft_red_ correlates well with chlorophyll content and peaks after Ft_blue_ (S1 Fig).

**Fig 3:**
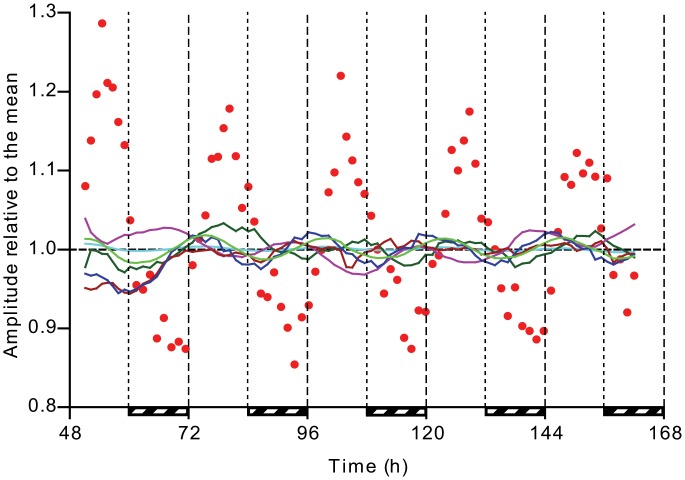
Relative amplitude of circadian oscillation. Shown are data from the free-running period following entrainment from the same culture as in Fig 1. Subjective ‘night’ in continuous light is indicated by striped bars. Each data point represents the average of a measured parameter in-between pump events. The time-weighted average of all data points after the first 24 h of continuous conditions (i.e. 84–162 h) was used to normalize each parameter to 1. All data points are plotted mid-cycle. Shown are growth rate (red circles), dissolved O_2_ (dark green line) and dissolved CO_2_ concentration (purple line), Ft_red_ (dark red line), Ft_blue_ (blue line), pH (cyan line) and the OD_680_/OD_720_ ratio (light green line).

The amplitude of each oscillation as a percentage change with respect to the mean is shown in Fig 3. For most parameters the amplitude is fairly small (within ±5% of the mean), except growth rate which oscillates between ±15% of the mean. The highest overall growth rate was seen in the light period of LD, which may be due to preparatory activity during the night which is either not done, or not possible, in continuous light [22].

**Fig 4:**
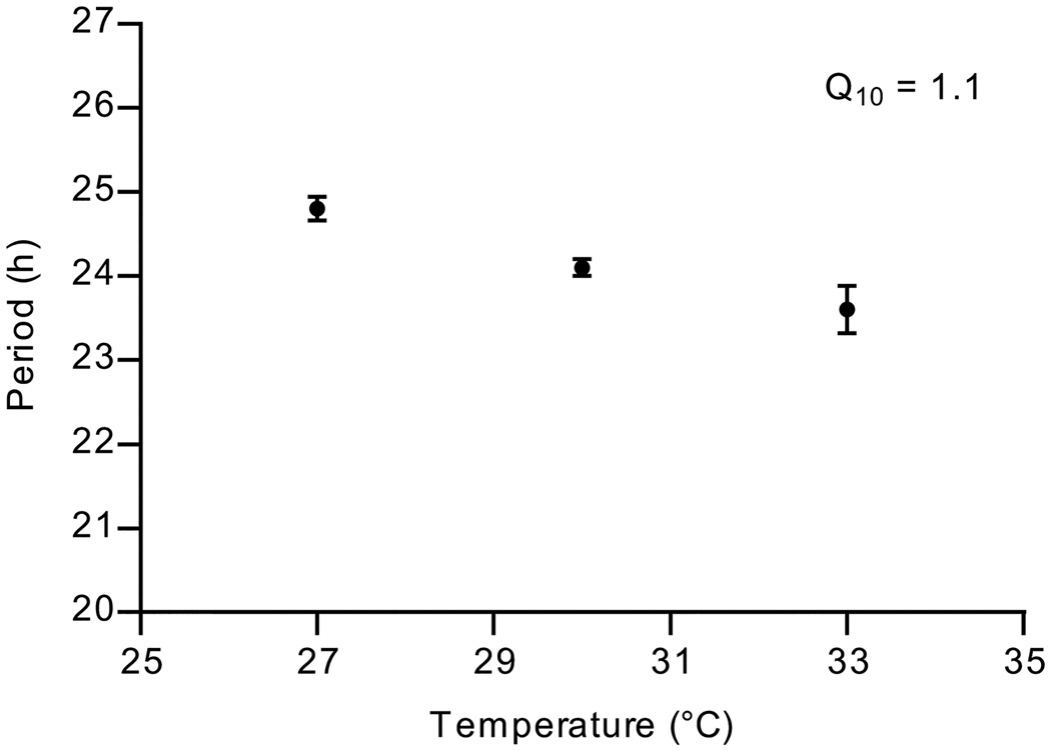
Temperature dependence of the free-running period of the circadian clock. The data are based on at least two independent experiments. For 30°C, three independent experiments were averaged. Error bars show the SD. The temperature dependence, Q_10_, of the period of the free-running circadian clock was calculated using a rate of 1 per period.

Another important property of a circadian clock is that a period length of approximately 24 h is maintained at different temperatures. Fig 4 shows that the oscillations in the free-running clock of *Synechocystis* fulfill this requirement, with a measured period length of 24.8 ±0.1 h at 27°C, 24.1 ±0.1 h at 30°C and 23.6 ±0.3 h at 33°C. This leads to a Q_10_ value of 1.1, which is similar to that of *Synechococcus* [6].

### Circadian oscillation in *Synechocystis* is robust and highly synchronized

After ~114 h in LL, turbidostat control was stopped to allow approximately a doubling of cell density before rapidly diluting the culture to within the chosen turbidostat thresholds (S2 Fig). The dashed line represents the fit to the last three periods before releasing turbidostat control, extended to the end of the experiment. Apart from the introduction of an upward trend in relative chlorophyll content, the period was unaffected by this perturbation. The feedback regulation of the relative chlorophyll content is visible as it converges on the value of the predicted rhythm. During this period of batch growth, the OD_680_/OD_720_ ratio increased beyond the predicted peak, but was instantly reduced upon dilution to a value well above that predicted by the fit (inset S2 Fig). This indicated non-linearity of the OD measurements caused the ratio to shift further than expected from the physiological response. To test whether or not this non-linearity affected the observed oscillations, the turbidostat thresholds were chosen such that it approximates a chemostat with almost constant dilution and OD_720_. In this period, between 350 h and 429 h, no growth rate data are available as a consequence of near-constant dilution, however, the lack of any effect on the OD_680_/OD_720_ ratio shows that in the chosen turbidostat range, non-linearity of the measured ODs did not affect the observed oscillation. It appears as if the circadian oscillation was slightly damped during the first two days of LL, but this can be explained by the smaller amplitude of the oscillation, compared to LD, due to lacking the night of LD to prepare for growth in the light. A relatively high peak in growth rate, OD_680_/OD_720_, etc., is always seen after a period of darkness.

Besides having a fully functional circadian clock that affects important physiological parameters like growth rate, chlorophyll synthesis and photosynthesis, it is striking that the free-running oscillation is maintained for almost three weeks, with no apparent damping in constant conditions. In *Synechococcus*, sustained oscillation is observed that can last for months [23,24]. This sustained oscillation, i.e. synchronization of the clock of each cell, was shown to be governed by a transcriptional-translational feedback loop, involving a KaiC-dependent promoter, located in-between *kaiA* and *kaiBC* [13]. Disruption of this feedback loop causes a rapid loss of synchrony between cells in a culture and is postulated to also be required at generation times shorter than 24 h to counteract the effective degradation of clock proteins by cell division and protein degradation [12,13]. Recent analysis of the transcriptome of *Synechocystis* revealed that a similar promoter is not present in the latter organism [17], yet oscillation is evidently highly synchronized and sustained in its cultures, with a generation time well below 24 h.

### Oxygen is required for chlorophyll synthesis

**Fig 5:**
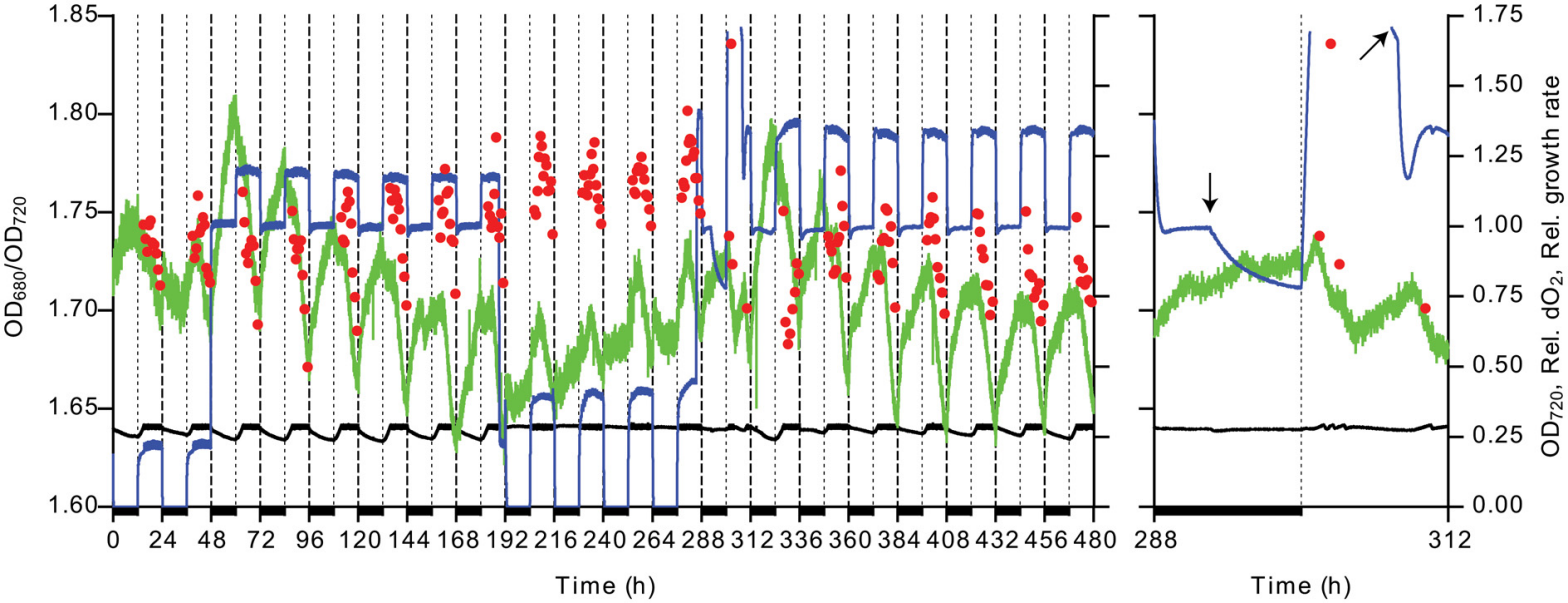
Chlorophyll content under 12h/12h dark/light regime in the presence and absence of O_2_. Shown are the OD_680_/OD_720_ (green line, left axis), OD_720_ (black line, right axis), dissolved O_2_ concentration normalized to the air-saturated concentration (blue line, right axis) and growth rate normalized to the median (red circles, right axis). The inset on the right shows an enlargement of the time window from 288 h to 312 h in which sparging was stopped and started as indicated by the arrows. The blue line shows a gap where the dissolved oxygen concentration exceeded the measuring range of the probe. Dark periods are indicated by a dark bar.

In Fig 1, a striking difference is visible between the chlorophyll content trace during darkness of the second ‘night’, compared to the subjective ‘night’ during free-running in LL. In darkness, no increase in relative chlorophyll content was observed, except for a short period after the onset of darkness during which oxygen was not yet fully removed, and rapid synthesis started when the light was turned on. Conversely, a sinusoidal pattern of net synthesis and degradation was seen in continuous light. This can be related to either the presence of light or the presence of oxygen, both of which are absent only in darkness, or a lack of energy in the absence of both. In biosynthesis of chlorophyll, the oxidation of protoporphyrinogen to protoporphyrin IX is oxygen-dependent in plants, but not in anoxygenic photosynthetic bacteria [25]. Interestingly, the homolog of the protein catalyzing this reaction that is present in *Synechocystis* suggests that an oxygen-independent bacterial variant is present instead of the oxygen-dependent plant and algal enzyme. In order to test the dependence of chlorophyll synthesis on O_2_, an LD rhythm was imposed in its presence and absence (Fig 5).

A clear difference, depending on the presence/absence of O_2_, was observed. In its absence, the chlorophyll content was approximately stable at a slightly higher level (i.e. OD_680_/OD_720_ ≈ 1.7 during the first 48 h) than when O_2_ was present in the dark (OD_680_/OD_720_ ≈ 1.65). In its absence, no change in chlorophyll content was observed in darkness, followed by rapid synthesis in the light. Furthermore, from the data displayed in Fig 5 it stands out that during the first night in the presence of O_2_ the highest net rate of synthesis of chlorophyll is observed, followed by a slow decrease in average chlorophyll content. The reverse trend was seen when the culture was subsequently switched back to nightly periods of anaerobiosis. The difference in average chlorophyll content may be explained by the involvement of feedback regulation, causing accumulation of biosynthetic intermediates in the absence of O_2_, and incidentally causing a slight overshoot in chlorophyll content with the onset of illumination and, consequently, the presence of O_2_. If the lack of O_2_ in the subjective night during LD is integrated into the regulation of chlorophyll synthesis, the presence of O_2_ in subjective night of LL may also account for the slightly larger amplitude seen in the first 12 h of LL.

## Conclusions

Here, we show that *Synechocystis* has an authentic circadian clock that fulfills all criteria and maintains a free-running circadian rhythm in continuous light with minimal damping that lasts well over two weeks, which is in contrast to earlier reports that showed either strongly damped or no oscillation in continuous illumination conditions [19,22]. Our approach of measuring multiple parameters simultaneously in a continuous culture with precisely defined growth conditions allowed for the detection of small amplitude oscillations and their underlying relation. Kondo & Ishiura remarked that often, damping of the oscillation is attributed to culturing conditions that do not allow for sustained oscillation to be assayed, rather than a property of the oscillator itself [26]. In the case of *Synechocystis*, the amplitude of all measurable oscillations—except growth rate—is small (Fig 3), which suggests that in most previous research, these oscillations were probably hidden in the noise, while growth rate was not precisely monitored. This interpretation is supported by the observation of Kucho et al. that growth rate oscillated rhythmically, despite identifying few oscillating transcripts. Circadian rhythms are sustained despite perturbations of growth conditions such as cell density (which also affects light availability), showing the robustness of the clock.

Even though the small amplitude may explain the low number of oscillating transcripts found in the works of Kucho and Beck, this does not account for the lack of sustained oscillation found by Beck et al. in the transcripts that were found to oscillate in LD. The culturing conditions (i.e. continuous growth) employed in the present study are similar, though unlikely to be identical, to those of Kucho et al., and very different from those of Beck et al., which may explain the similarities and discrepancies between those results and the ones reported here. However, it begs the question of why the conditions chosen by Beck et al. do not allow for a sustained free-running circadian rhythm and why no gradual damping was observed as expected in the case of unsynchronized oscillators. A possible explanation is that very slow growth, as observed by Beck et al., disables the clock under continuous light which is supported by earlier observations of van Thor [27].

We chose a background of N_2_ to mimic mass culture conditions in which anaerobic conditions may prevail during the night (e.g. when CO_2_ from an anaerobic digester or industrial off-gases supply the CO_2_), which is a major difference with previous work. However, the data from Fig 5 indicate that anaerobiosis in the night does not influence the circadian rhythm and further emphasizes the robustness of the circadian clock in *Synechocystis*. Additionally, by utilizing a photobioreactor with precisely controlled growth conditions and measurements rather than bioluminescence, it is possible to follow oscillations over time in anaerobic and micro-aerobic conditions.

Precisely because of the sensitivity of the on-line measurements, it is critical to exclude that periodic variations in external conditions (e.g. room temperature) are the actual cause of the observed oscillation. No such effect was found. Neither starting entrainment at various points of the actual day or, alternatively, completely covering the PBR with dark cloth, nor changing pump rate and turbidostat range (Fig 5) had any measurable effect on the period of oscillation or the phase relationship with the zeitgeber.

The sustained, remarkably undamped oscillation shown here raises many questions. It was convincingly shown in *Synechococcus* that a TTR loop is required to maintain synchrony between cells in a (fast-) growing culture [12,13], but the lack of a promoter in-between *kaiA* and *kaiB1C1* makes this particular TTR unlikely to be the case in *Synechocystis* [17]. Furthermore, transcript levels of *kaiAB1C1* were shown to accumulate to similar amounts in the same phase [22]. The specific *Synechocystis* strain used in the study of Beck et al. does differ from the strain used in this study. However, they reported no clock-related mutations compared to the originally sequenced strain, which is a close relative of our strain [28].

An explanation might be found in the additional homologs of the clock genes that were found in *Synechocystis*, though this seems unlikely. Recently, Dörrich et al. showed impaired growth in a Δ*kaiAB1C1* mutant under LD, whereas a Δ*kaiC3* mutant had a phenotype identical to that of the wild type [29]. Additionally, *kaiB2C2* could not be deleted which indicates they are not involved with the core oscillator and may be an unrelated operon with homology to *kaiBC* such as found in many non-cyanobacteria [10]. Earlier work showed that KaiC2 and KaiC3 do not interact or co-purify with KaiA, further suggesting that there is no link to the clock components [30]. Even though the circadian rhythm in *Synechocystis* seems remarkably similar to that of *Synechococcus*, its regulation appears to differ in a critical component and is of great interest for further research. Intercellular communication with respect to the clock has been shown not to occur in *Synechococcus* [23], but in *Cyanothece* sp. ATCC 51142, spontaneous rhythms were observed in late stationary phase or when exchanging fresh medium for spent medium [31].

The presence of oxygen appears critical for the synthesis of chlorophyll. Despite having homologs of oxygen-independent enzymes for various steps in the pathway [25,32], it is evident that chlorophyll synthesis in *Synechocystis* depends on the presence of O_2_, which may have implications for large-scale photobioreactors employing O_2_-poor gas mixtures.

## Materials and Methods

### Growth conditions

Wild type *Synechocystis* sp. PCC6803 (a glucose-tolerant derivative, obtained from D. Bhaya, Department of Plant Biology, Carnegie Institution for Science, Stanford, USA) was grown in modified BG-11 medium [33] in a photobioreactor (model FMT 150.2/1000, Photon Systems Instruments, hereafter called PBR) in continuous light prior to the start of an experiment. BG-11 was modified along the lines of YBG-11, by replacing ammonium ferric citrate and citrate by 15 μM FeCl_3_·6H_2_O and increasing EDTA Na_2_·2H_2_O to 15 μM [34]. Na_2_CO_3_ was omitted altogether as CO_2_ was supplied either by sparging the PBR with CO_2_ or by separate addition of NaHCO_3_ to batch cultures. This was done to avoid iron precipitation in the medium-supply vessel during prolonged cultivation and had no measurable effect on growth rate or yield.

The PBR is a temperature-controlled flat-panel type photobioreactor with a working volume of approximately 960 mL, which is illuminated by an LED panel from one side and is described in greater detail in [35]. The lid of the PBR accommodates a combined pH/temperature probe, a Clark-type dissolved O_2_ (dO_2_) probe and a potentiometric dissolved CO_2_ (dCO_2_) probe (all Mettler-Toledo). The integrated densitometers measure optical density (OD) at 720 and 680 nm and were used to control cell density (OD_720_) and measure relative chlorophyll content (OD_680_/OD_720_). Measurements of all sensors were recorded at one-minute intervals. The LED panel contains red (636 nm, 20 nm full width at half maximum) and blue (445 nm, 18 nm full width at half maximum) LEDs, set to an intensity of 100 and 25 μmol photons/m^2^/s, respectively. This light intensity was measured outside the PBR, at a central point opposite the LED panel with the culture vessel in place and filled with fresh medium.

A constant supply of CO_2_ was provided by sparging the medium with 0.5% CO_2_ (v/v) in N_2_, mixed by a gas mixing system (GMS150, Photon Systems Instruments). The gas flow to the PBR was 150–500 mL/min depending on the sparging characteristics of the particular PBR, controlled by a mass flow controller (Smart Mass Flow Model 5850S, Brooks Instruments). The PBR was inoculated to approximately OD_730_ = 0.1, as measured on a bench-top photospectrometer (Lightwave II, Biochrom; OD_730_ = 1 ≈ 10^8^ cells/mL) using a pre-culture routinely cultivated in modified BG-11 medium supplemented with 5 mM NaHCO_3_ at moderate illumination (~30 μE/m^2^/s cool white fluorescent light) [36]. The culture was subsequently grown to steady state, defined as a constant growth rate and dO_2_. The OD_720_ as measured by the PBR is calibrated to the bench-top photospectrometer OD_730_ to maintain the OD_730_ at approximately 0.6 in turbidostat mode. The OD_720_ thresholds for the turbidostat were chosen ±2% of the target OD_720_. Upon inoculation of the PBR with growing *Synechocystis* cells, dO_2_ first peaks and subsequently declines, as the culture grows to the target optical density of 0.6. This, and an increase in growth rate at higher light intensity, indicated that these were light-limited growth conditions for the organism. Entrainment of the circadian clock to a 12 h/12 h light/dark (LD) rhythm was done in a 48 h period and was followed by a period of free-running in continuous light (LL). Temperature was kept constant at 30 ±0.2°C.

### Data analysis

The cell density measurements per growth cycle (i.e. OD_720_ in-between the set thresholds) were used to quantify the specific growth rate of a given cycle using Eq (1),

$$\mu \;=\;(\Delta \text{ln}({OD}_{720}))/\Delta t$$ (1)

In order to account for noise spikes that disturb the cycle (e.g. causing the pump to start early), the average was taken over four time periods, each one minute further away from the thresholds into the cycle. The dilution rate could not be used as the pump speed depended on the condition of the pump tubing, which degrades over time. The period of the oscillation observed during free running was analyzed using non-linear regression and the solver of Excel (Microsoft) described in [37]. The OD_680_/OD_720_ ratio as proxy for chlorophyll content was fitted to a sine function with parameters for amplitude, period, phase shift, offset and slope. This ratio is unaffected by the turbidostat regime and could be used without further processing. A parameter for slope was added to account for a trend in the data. Initial parameters were derived from the data to obtain an approximate fit which was further refined by the solver. The first 36 h of continuous light were ignored, because of the difference in amplitude between LD and LL. For the correlation of growth rate to other measured parameters, Pearson’s correlation coefficient was calculated using the average of a given parameter per growth cycle. This was done by comparing the full range of cycles at phase shift 0. A phase shift was then obtained by selecting a subset of cycles such that the start of the compared parameter was shifted by the desired number of cycles with respect to the growth rate.

## Supporting Information

S1 Dataset Raw data of entrainment and free-running at 30°C. Output of the photobioreactor with a *Synechocystis* sp. PCC6803 culture entrained to a 12/12 h dark/light rhythm followed by free-running. The data are displayed in Fig 1 and S2 Fig.
(XLSX)

S1 Fig Pearson’s correlation of growth rate with other parameters at 33°C. At 33°C, fluorescence emission from phycobilisome excitation (red diamonds) better correlates with growth rate, but no difference is seen for relative chlorophyll content (OD_680_/OD_720_, light green circles) and fluorescence emission from chlorophyll excitation (blue line with crosses). A value of 1 indicates perfect positive linear correlation, 0 the absence of correlation and -1 perfect negative correlation. Note that the phase shift is obtained from sliding along the data points (i.e. growth/dilution cycles), which do not all represent the same time interval due to differences in growth rate. Shown further: pH (cyan squares), dissolved O2 (green upward triangles) and dissolved CO2 concentration (purple downward triangles).
(EPS)

S2 Fig Continued oscillation despite small perturbations in continuously grown *Synechocystis* sp. PCC6803 in a photobioreactor in turbidostat mode. This is the same data set as in Fig 1, showing growth rate per dilution cycle (red circles, left axis), the OD_680_/OD_720_ ratio (green line, right axis) as a proxy for chlorophyll content and the fit thereof (dashed line, right axis), now extrapolated to the end of the experiment. Between 161.5 and 178.9 h, turbidostat control was switched off to perturb the culture (inset). Between 350 and 428 h, the turbidostat thresholds were chosen such that a chemostat with constant dilution was approximated, causing OD_720_ to be kept almost constant.
(EPS)
